# Case report: Familial foveal retinoschisis caused by *CRB1* gene mutation in a family with recessive inheritance

**DOI:** 10.3389/fmed.2023.1220075

**Published:** 2023-08-11

**Authors:** Shu Liu, Yue Ren, Di Wang, Dan Xiao, Zhuang Li, Dan Xu, Yan Sun, Zhuoshi Wang, Jijing Pang

**Affiliations:** ^1^Shenyang He Eye Specialist Hospital, Shenyang, China; ^2^Liaoning Provincial Innovation Center of Ophthalmology, Shenyang, China; ^3^Shenyang Weijing Biotechnology Co., Ltd., Shenyang, China; ^4^Institute of Innovation Research for Precision Medical Treatment, He University, Shenyang, China

**Keywords:** *CRB1* gene, recessive mutation, familial (isolated) foveal retinoschisis, genetic testing, clinical manifestations

## Abstract

X-linked retinoschisis is more common in male children and rare in females. Clinically, male patients mainly present with early onset visual impairment or vision loss, and retinal retinoschisis due to division of the inner retina. We report a long-term observation of a female patient with familial foveal retinoschisis (FFR) caused by *CRB1* gene with complex heterozygotic mutation. The initial symptoms of the female patient reported in this study were very similar to some early manifestations of X-linked retinoschisis (XLRS) caused by *RS1* mutations involving macular fovea. However, as time going on, the splitting height at retinal fovea of FFR gradually decreased, and the splitting extent at retinal fovea of FFR gradually decreased.

## Introduction

Retinoschisis can occur in the fovea or peripheral retina, and generally includes congenital/hereditary, acquired (age-related) and secondary types (1), with significant differences in etiology, site of onset, and clinical manifestations. X-linked retinoschisis is more common in male children and rare in females. Clinically, male patients mainly present with early onset visual impairment or vision loss, and retinal retinoschisis due to division of the inner retina. X-linked retinal retinoschisis (XLRS) are usually caused by mutations in retinoschisin 1 located in Xp22.13 (2), except for the rare cases of autosomal dominant retinal retinoschisis (3). In general, women are mostly carriers except for homozygous variants caused by conspecific marriage (4), and most female carriers have no significant visual function and electroretinogram (ERG) (5). However, a female patient with retinoschisis has been reported to have heterozygous variants of *RS1* gene (6).

Hereditary retinal diseases caused by *CRB1* gene variants may have different clinical manifestations. Online Mendelian Inheritance In Man (OMIM) includes Leber congenital amaurosis (LCA), retinitis pigmentosa (RP), early-onset cone-rod dystrophy (EOCRD) and pigmentary paravascular chorioretinal atrophy (PPCRA) (7, 8). In 1977, Lewis et al. (9) first described Familial Foveal Retinoschisis (FFR), and reported that three female patients in a family presented with mild visual loss. FFR was first reported by Vincent et al. (10) as a recessive hereditary eye disease caused by *CRB1* gene mutation.

The purpose of this study was to identify the pathogenic genes and clinical characteristics of female patients diagnosed with retinal retinoschisis in the outpatient department, and to improve the pathogenic gene profile and the natural course of the disease.

## Materials and methods

### Patients

The study was approved by the Ethics Committee of Shenyang He Eye Specialist Hospital (IRB (2016) K001.01). Written consent forms were obtained from all participants or their guardians. A Han female patient with retinoschisis diagnosed in the outpatient department and three members of her family with normal phenotype were enrolled in the He Eye Specialist Hospital, Shenyang, China.

**Figure 1:**
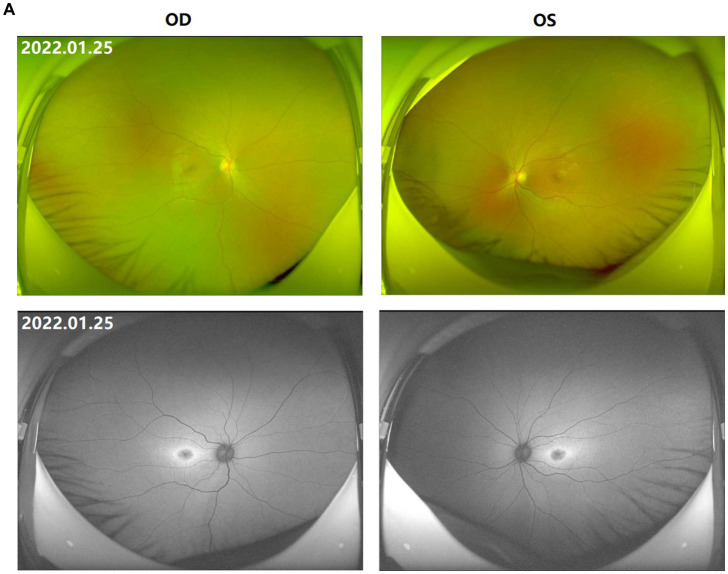

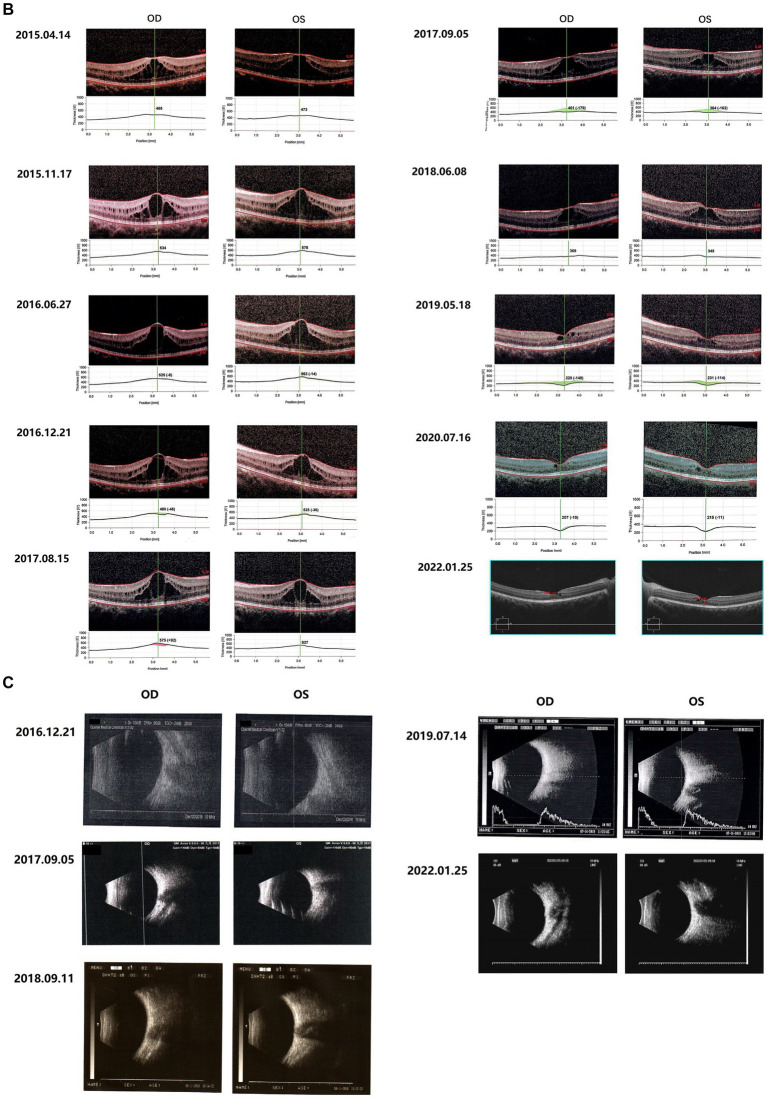
Fundus images of the patient (III-1). **(A)** Binocular scanning laser ophthalmoscope (SLO) and fundus autofluorescence (AF) in January 2022: SLO showed that the macular area of the fundus was dark and no foveal reflection was found in both eyes, AF examination showed low autofluorescence in the macular region and annular high autofluorescence around the macula. **(B)** OCT images around the macular area in Bilateral eyes from 2015 to 2022: OCT showed that: the thickness of splitting central fovea was 465 μm in the right eye and 473 μm in the left eye on April 14, 2015; the foveal thickness was 534 μm in the right eye and 578 μm in the left eye on November 17, 2015; the foveal thickness was 525(−8) μm in the right eye and 563(−14) μm in the left eye on June 27, 2016; the foveal thickness was 480(−48) μm in the right eye and 525(−36) μm in the left eye on December 21, 2016; the foveal thickness was 575(+92) μm in the right eye and 527 μm in the left eye on August 15, 2017; the foveal thickness was 401(−179) μm in the right eye and 364(−162) μm in the left eye in September 5, 2017; the foveal thickness was 369 μm in the right eye and 345 μm in the left eye in June 8, 2018; the foveal thickness was 220(−148) μm in the right eye and 231(−144) μm in the left eye in May 18, 2019; the foveal thickness was 207(−15) μm in the right eye and 215(−11) μm in the left eye in July 16, 2020; the foveal thickness was 166 μm in the right eye and 167 μm in the left eye in January 25, 2022; the specific check time is marked in the upper left corner of the picture; the values in parentheses represent the difference of average thickness at foveal area compared with the last examination. **(C)** Results of B-ultrasound examination of both eyes from 2016 to 2022: B-ultrasound showed mild vitreous opacity in both eyes; the posterior pole of both eyes was thickened and rough on December 21, 2016 with mild vitreous opacity in both eyes after September 5, 2017.

### Clinical investigation

In this study, the family survey research method was used. Detailed records of the patient’s medical history and family history and the history of marriage between first Cousins. Ophthalmic examinations were performed on the enrolled members, including best corrected visual acuity, slit-lamp microscopy of the anterior segment, color vision examination, B-ultrasound examination, visual field examination, scanning laser ophthalmoscope (SLO) and autofluorescence (AF) examination, optical coherence tomography (OCT) examination, visual evoked potential (VEP) and electroretinogram (ERG) examination.

### Genetics analysis

Whole exome sequencing was used in this study. Pathogenicity rating of variants and data interpretation rules were based on the American College of Medical Genetics and Genomics (ACMG) guidelines and the recommendations of sequence variation Interpretation (SVI) expert group in ClinGen (11–14). Excluding variants with a mutation frequency greater than 1% in the 1000 Genomes, ExAC, and gnomAD databases, and excluding non-functional variants (such as sense variants and non-coding region variants). After comprehensive consideration of pathogenicity prediction (SIFT, Polyphen2, CADD and other software), clinical symptom control, related disease database query and literature reference, candidate gene variants were found for family verification.

## Results

### Clinical phenotypes

**Figure 2:**
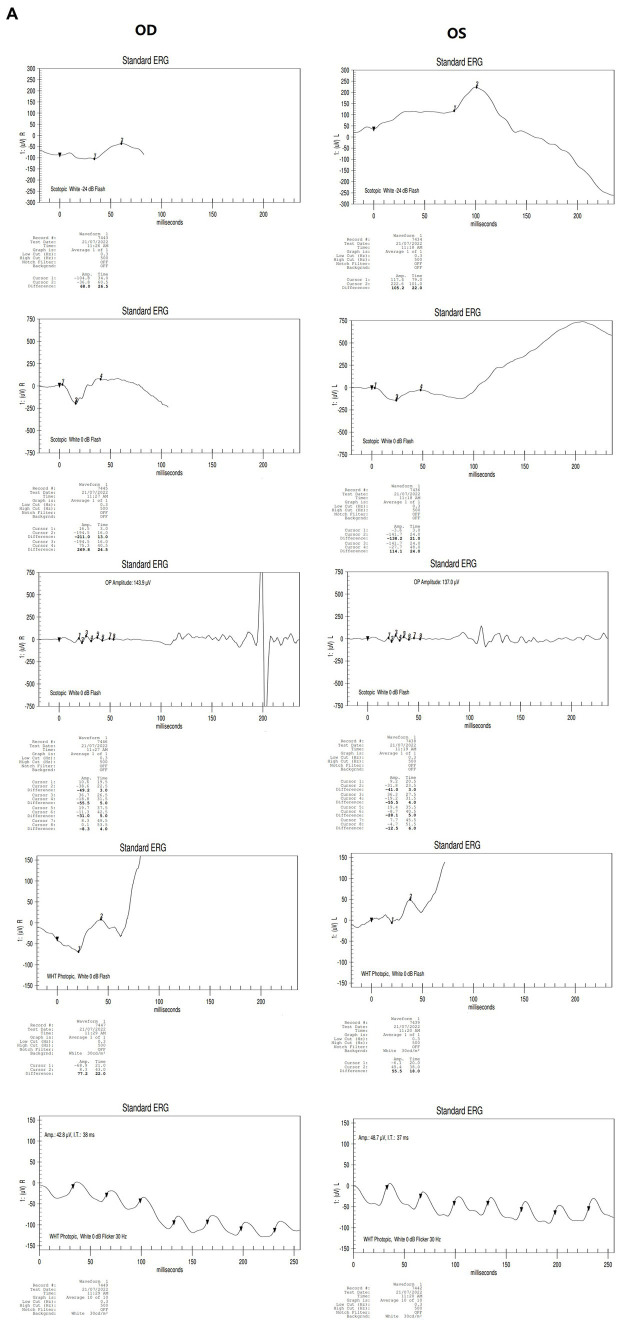

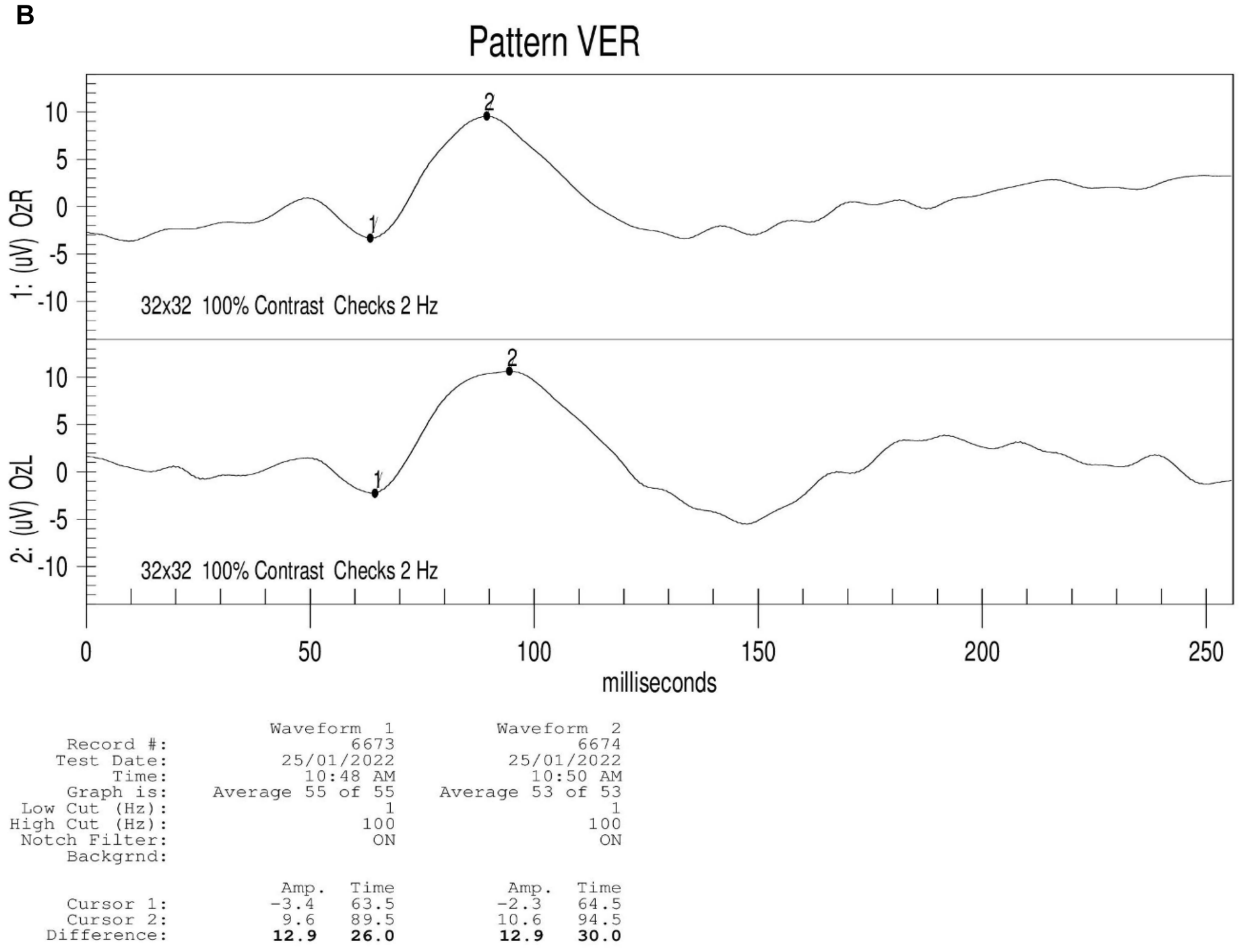

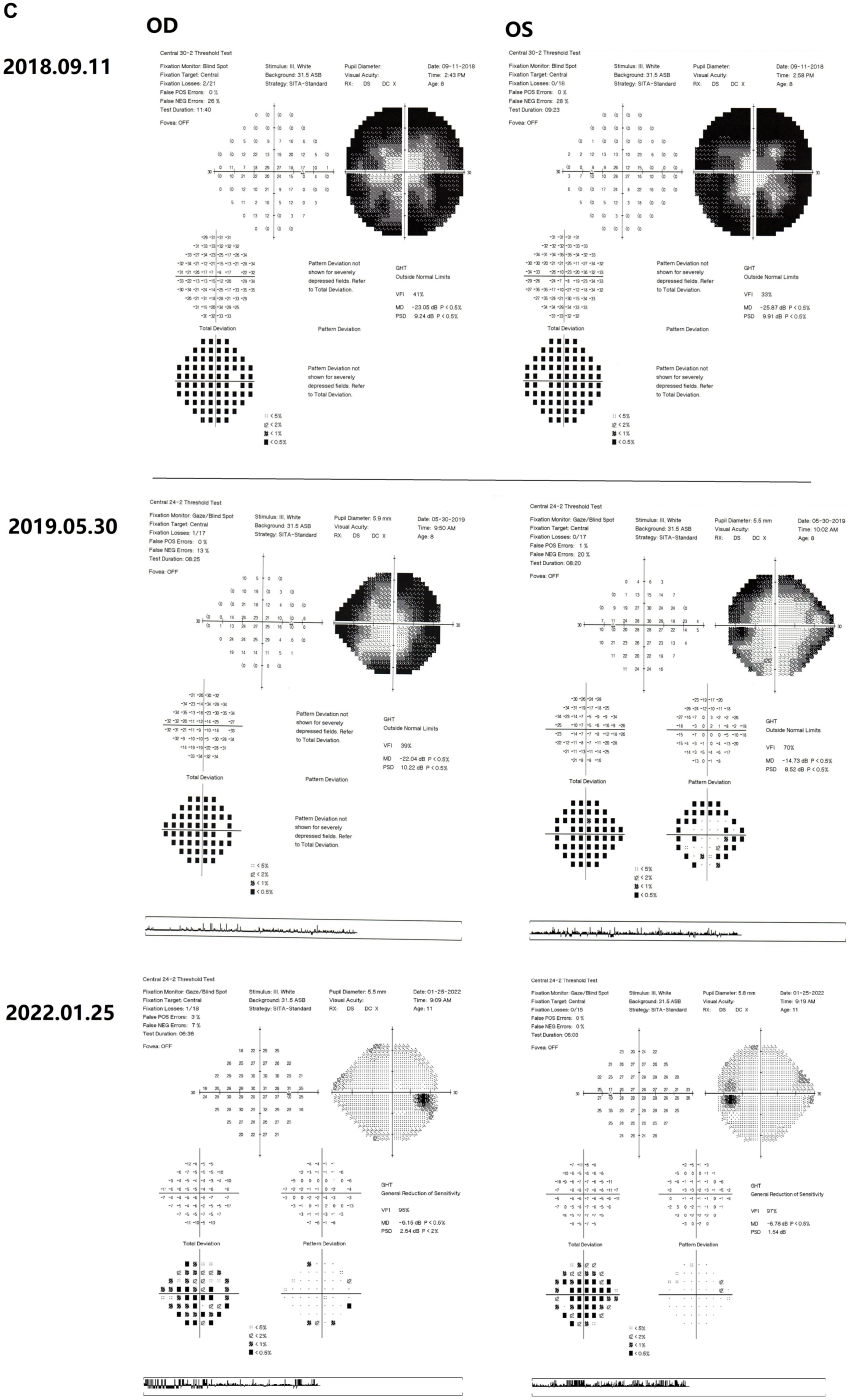
Results of binocular ERG **(A)** and VEP **(B)** in 2022; Binocular visual field examination **(C)** in patient from 2018 to 2022. **(A)** The results of ERG showed that OD (oculus dexter): scotopic white 24 dB (decibel) flash: amplitude = 68 μV (microvolt), time = 26.5 ms; scotopic white 0 dB flash: a wave = −211.0 μV, time = 13 ms; b wave = 269.8 μV, time = 24.5 ms; OP amplitude = 143.9 μV; white photopic:white 0 dB flash: amplitude = 77.2 μV, time = 22 ms; white 0 dB flicker 30 Hz: amplitude = 42.8 μV, time = 38 ms. OS (oculus sinister): scotopic white 24 dB Flash:amplitude = 105.2 μV, time = 22 ms; scotopic white 0 dB flash: a wave = −138.2.0 μV, time = 21 ms; b wave = 114.1.8 μV, time = 24 ms; OP amplitude = 137.0 μV; white photopic:white 0 dB flash:amplitude = 55.5 μV, time = 18 ms; white 0 dB flicker 30 Hz: amplitude = 48.7 μV, time = 37 ms. **(B)** The results of VEP showed that amp = 12.9 μV, time = 26.0 ms (millisecond) in the right eye and amp = 12.9 μV, time = 30.0 ms in the left eye. **(C)** The results of visual field examination (central 30–2 threshold test) showed that the VFI (vision field index) of the right eye was 41% and the VFI of the left eye was 33% on September 11, 2018. The results of visual field examination (central 24–2 threshold test) showed that the VFI of the right eye was 39% and the VFI of the left eye was 70% on May 30, 2019. The results of visual field examination (central 24–2 threshold test) showed that the VFI = 95% in the right eye and 97% in the left eye on January 25, 2022.

The proband (III-1) was a 12-year-old girl. At the age of four in 2014, her family found that the child had poor vision, and she was diagnosed with bilateral retinoschisis (macular area), which was considered as congenital retinoschisis. The patient was admitted to the outpatient department of our hospital in January 2022, VOD (visio, oculus dexter):0.8 (1.0* + 1.50DS = −1.75 DC*175°); VOS (visio, oculus sinister):0.3 (1.0^–2^* + 2.00DS = −2.75 DC*180°). Slit-lamp examination of the anterior segment of both eyes revealed no abnormalities. SLO showed that the macular area of the fundus was dark and the foveal reflection was not reached in both eyes (Figure 1A). OCT showed that the foveal thickness was 166 μm(micrometre) in the right eye and 167 μm in the left eye (Figure 1B). B-ultrasonography revealed vitreous opacities (small amounts) in both eyes (Figure 1C). Binocular flash ERG showed a mild to moderate decrease in rod response amplitude, a decrease in maximum mixed response amplitude, a decrease in negative b wave (b wave was more obvious than a wave), a normal oscillatory potential, and a slight decrease in cone and 30 Hz flicker response amplitude (Figure 2A). Color vision and VEP studies were unremarkable (Figure 2B). The results of visual field examination showed that the visual field of both eyes had improved compared with the visual field before May 30, 2019 (Figure 2C).

Since 2015, the patients were regularly reviewed for macular thickness (horizontal tomographic OCT, the vertical distance from the highest retinal neuroepithelial layer to the retinal pigment epithelial layer). OCT showed that the degree of foveal retinoschisis gradually increased in the early stage. The results of OCT examination in September 2017 showed that the foveal retinoschisis decreased from the height of the last examination in August 2017. And compared with August of the same year, the thickness of foveal retinoschisis decreased significantly, and the foveal retinal schesis gradually developed into parafoveal cystoid degeneration, and the cyst-like vesicles gradually decreased. The morphology of the fovea returned to almost normal in 2020 (Figure 1B).

### Gene sequencing results

**Figure 3:**
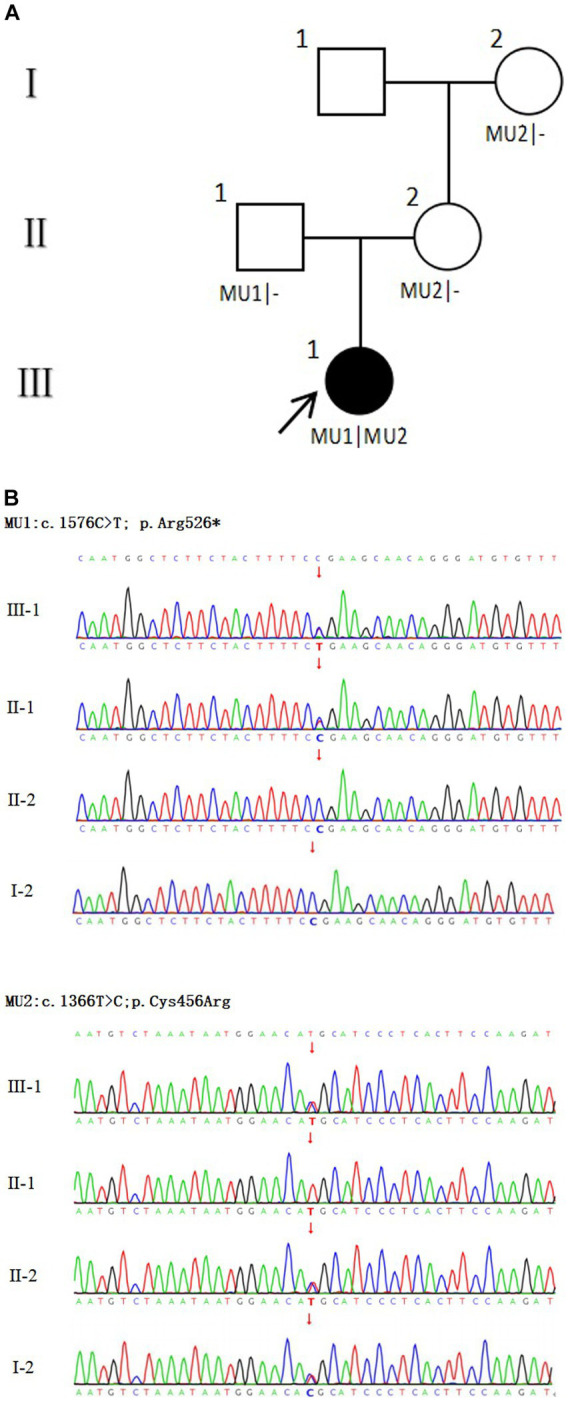
Family diagram and *CRB1* gene mutation. **(A)** Family chart ⬤ female patient; ▢ normal male; ◯ normal female; ↗ proband; **(B)** Validation diagram of *CRB1* mutant generation.

In this study, two mutations of c.1576C > T; p. Arg526* (NM_201253.3) and c.1366 T > C; p. Cys456Arg (NM_201253.3) were detected in the proband *CRB1* gene. *CRB1* gene mutation is autosomal recessive inheritance. c.1576C > T is a known pathogenic mutation, which changes the corresponding codon into a stop codon and leads to changes in protein function. This mutation is inherited from the father (II-1). c.1366 T > C was a novel mutation, which had not been found in ExAC, 1000G and gnomAD databases. The results of multiple statistical methods (REVEL) showed that the variant caused harmful effects on the gene or gene product. This mutation was detected in the samples of mother (II-2) and grandmother (I-2), and it constituted a complex heterozygous with the mutation locus c.1576C > T. According to The American College of Medical Genetics and Genomics, the mutation was likely pathogenic. No other variants associated with retinal disease were detected. The family diagram is shown in Figure 3.

## Discussion

Crumbs homolog 1 (*CRB1*) gene encodes a transmembrane protein similar to Drosophila crumbs protein, located at 1q31.3 (OMIM:604210). Its transcript contains many EGF-like domains and laminin G domains. CRB1-A and CRB1-B are expressed in Muller cells and photoreceptor cells of the retina. Transcript A is the most abundant and located at the top of Muller cells during development. Transcript B is dominant in the adult retina and is localized in the inner and outer segments of photoreceptors (15). It can interact with membrane palmitoylated protein 5 (MPP5) to maintain cell apical polarity and mediate intercellular adhesion (16). In this case, the mutation site of the patient was concentrated in the exon 6 region, and the mutation site c.1366 T > C (p. Cys456Arg) was a new mutation site, which had not been reported before. According to the protein structure prediction, the mutation site was located in the EGF-like domain. The mutation may affect the protein structure and the interaction between proteins. The other mutation, c.1576C > T (p. Arg526*), is a known pathogenic mutation located in the laminin G domain. This mutation causes protein truncation and changes its function, thus affecting cell adhesion and signal transduction. Therefore, it may affect the interaction between retinal cells, resulting in the formation of retinoschisis or capsule-like structures between retinal layers.

In 1977, Familial Foveal Retinoschisis (FFR) was first described by Lewis et al., who reported that three female patients in a family presented with mild visual loss and binocular foveal retinoschisis changes similar to XLRS (9). OCT results of FFR patients show typical foveal retinoschisis or cystoid uplift in the fovea, and the peripheral retina is generally normal (9, 17, 18). Among the cases reported in the previous literature, 11 were female patients and only one was male (9, 10, 17–19). FFR is a recessive hereditary eye disease caused by *CRB1* gene mutation, which was first reported by Vincent et al. (10). Considering the recessive inheritance and sporadic nature of the disease, Kabanarou et al. also referred to this disease as isolated foveal retinoschisis (18). Patients with *CRB1* gene variants usually present with cystoid macular changes before the age of 30 (20, 21). In addition, over time, the patient’s fundus changes from foveoschisis to cyst-like degeneration, and finally develops into atrophic macular degeneration (22, 23). In most cases, this is the natural course of FFR caused by mutations in the *CRB1* gene. The retina of patients usually begins to change around the age of 25–30, and the retina develops from foveal retinoschisis to macular or foveal cyst-like degeneration. However, the patient’s retina has entered the stage of cyst-like degeneration at the age of 12. The OCT results suggested that the thickness of the foveal retinoschisis had been increasing since the record was made in 2015 until August 2017, before the injury occurred. The day after the patient fell and hit the ground on her face, OCT showed that the retinoschesis relieved a lot and gradually disappeared in 2 years with only mild parafoveal cystoid macular degeneration. We cannot show any direct evidence that the quick relief of foveal retinoschesis after accident is caused by the force on her face, although it is possible.

In this case, the female patient has been diagnosed with congenital retinoschisis, which may be due to the fact that the early symptoms of foveoschisis are basically the same as the manifestations of foveoschisis in XLRS. However, the rapid reduction of foveoschisis after trauma, the absence of *RS1* gene mutation, and the female sex of the patient are contrary to the common clinical manifestations of XLRS. The clinical manifestations of this patient are also different from the common clinical manifestations caused by *CRB1* gene mutation. It has been reported that FFR caused by *CRB1* gene may have different ERG manifestations (10). However, the ERG results of XLRS patients usually show that the a-wave amplitude is basically normal or slightly decreased, the b-wave amplitude is decreased, and the b/a ratio is decreased. It is particularly noteworthy that although the parafoveal foveoschisis can also present as a “bridge-like” or “spoke-like” change in the early stage of FFR patients; However, the foveal retinoschisis is mainly cystoid retinoschisis. However, on the second day after the external force collision, the retinoschisis in the fovea, which had been relatively stable, was rapidly relieved, and the vision was improved. Even if we cannot be sure that the impact of the face on the ground is the root cause of the apparent reduction of foveal retinoschisis the next day, we cannot exclude the possibility that the cystoid retinoschisis retracted in a short time due to the release of the fluid in the raised capsule from the small tear by the impact of trauma. Because we have also encountered the phenomenon of retinal flattening at the foveal retinoschisis after trauma in patients with XLRS. Although the natural course of FFR also transitions from foveal retinoschisis to cystoid degeneration, it is likely that the collision accelerated the rate of change in this patient’s foveal retinoschisis. Although there is no direct scientific evidence to prove that trauma can accelerate the process of the disease, external factors may have a certain impact on the disease (24). At present, some researchers use carbonic anhydrase inhibitors (CAI) to treat retinoschisis or cystoid macular edema caused by *CRB1* gene mutation (25, 26). It is also meaningful to take preventive measures to protect macula in atrophy stage.

## Conclusion

**Figure 4:**
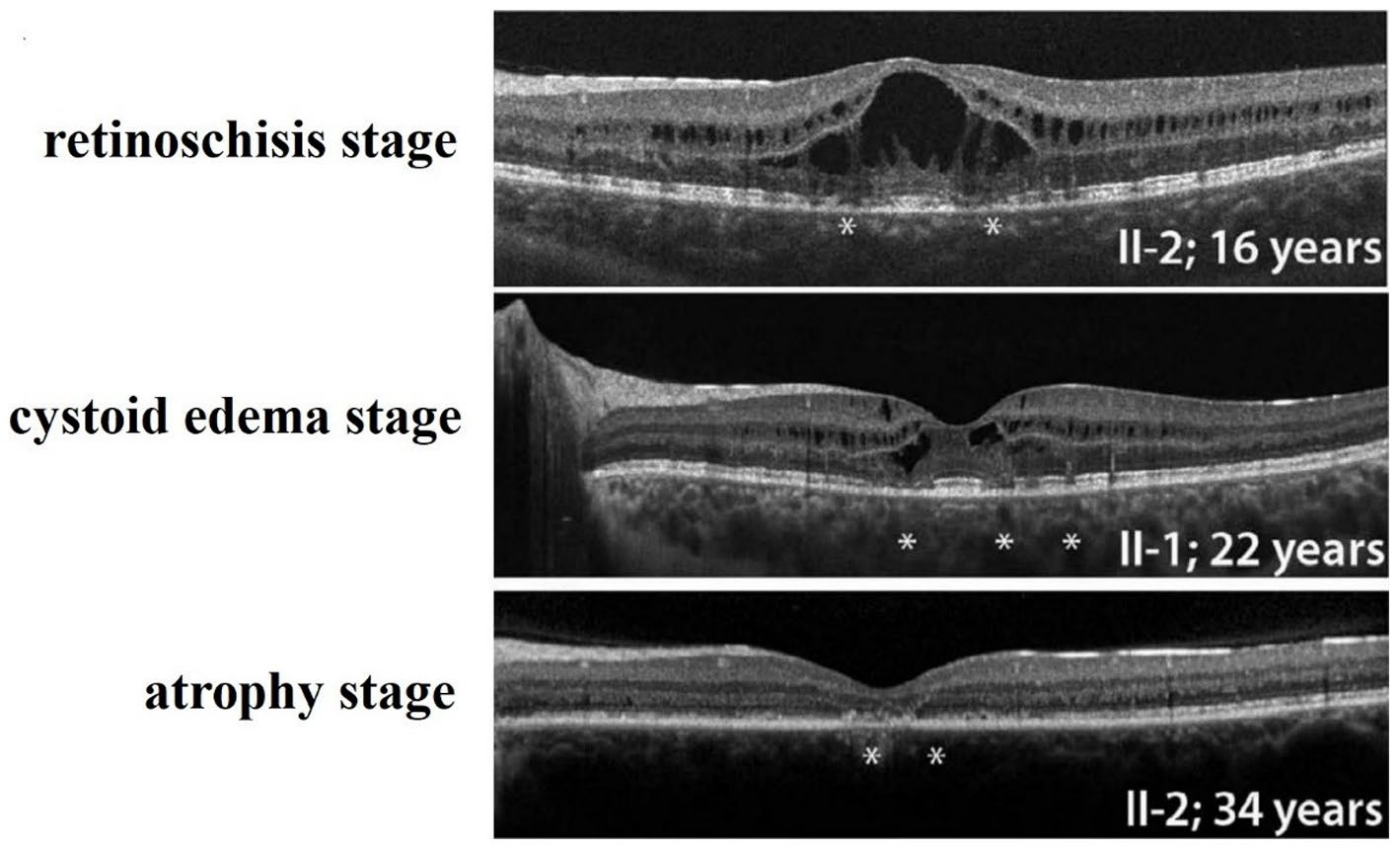
Three stages of Familial Foveal Retinoschisis, (PMID: 27258436). *Marked areas of localized destruction of the photoreceptor IS, OS, and ELM as shown by SD-OCT.

In summary, hereditary retinal diseases caused by mutations in the *CRB1* gene may have different clinical phenotypes, Including Leber congenital amaurosis (LCA), retinitis pigmentosa (RP), early onset cone-rod dystrophy (EOCRD), paravertebral chorioretinitis pigmentosa (PPCRA) (7, 8), and less severe isolated foveal retinoschisis, which vary in onset time and symptoms. The retinoschisis caused by *CRB1* gene mutation reported in this case may be the mild phenotype, the least impact on vision, and the best prognosis in the spectrum of retinal diseases caused by *CRB1* gene. The course of the disease can be divided into three stages: the retinoschisis stage, the cystoid edema stage, and the atrophy stage (Figure 4) (10). Whether the environment, drugs and external forces can affect or change the prognosis of this disease has not yet been confirmed by evidence-based medicine. Moreover, whether the cause of these different clinical manifestations caused by *CRB1* gene mutation is related to the relevant special sites of *CRB1* mutation remains to be elucidated. It is particularly noteworthy that although the exact cause of familial foveal retinoschisis caused by *CRB1* gene variants is unknown, the majority of patients with familial foveal retinoschisis are female. This is an important clinical difference between the early stage of this disease and sex-linked retinoschisis caused by *RS1* gene mutation.

## Data availability statement

The original contributions presented in the study are included in the article/supplementary material, further inquiries can be directed to the corresponding author.

## Ethics statement

The studies involving human participants were reviewed and approved by the Ethics Committee of Shenyang He Eye Specialist Hospital. Written informed consent to participate in this study was provided by the participants’ legal guardian/next of kin. Written informed consent was obtained from the minor(s)’ legal guardian/next of kin for the publication of any potentially identifiable images or data included in this article.

## Author contributions

JP conceptualized and designed the study and analyzed the cases and revised the manuscript. JP, YR, and SL recruited the patients. DW and DXi collected the data. JP, SL, and YR obtained patients’ consent, examined, and followed-up the patients. SL and YR analyzed the genetic testing results, reviewed the literature, and wrote the manuscript. ZL, DXu, YS, and ZW provided administrative and technical support. All authors approved the final manuscript.

## Funding

This work was supported by the National Natural Science Foundation of China (81970840) and the Shenyang Science and Technology Project (No.20-301-4-00).

## Conflict of interest

DXu and JP were employed by Shenyang Weijing Biotechnology Co., Ltd.

The remaining authors declare that the research was conducted in the absence of any commercial or financial relationships that could be construed as a potential conflict of interest.

## Publisher’s note

All claims expressed in this article are solely those of the authors and do not necessarily represent those of their affiliated organizations, or those of the publisher, the editors and the reviewers. Any product that may be evaluated in this article, or claim that may be made by its manufacturer, is not guaranteed or endorsed by the publisher.
